# Supplementary material for: HIVprotI: an integrated web based platform for prediction and design of HIV proteins inhibitors
Source: J Cheminform. 2018 Mar 9;10:12. doi: 10.1186/s13321-018-0266-y (PMC5845081; doi:10.1186/s13321-018-0266-y)
Supplement: Supplementary file 2 — Additional file 2. Source code of HIVProtI web server. [file 13321_2018_266_MOESM2_ESM.zip › HIVprotI_Source-code/hivprotI/weka-3-6-6/doc/weka/gui/beans/TextEvent.html]

TextEvent


---


|  |  |  |
| --- | --- | --- |
| **Overview** \| **Package** \| **Class** \| **Tree** \| **Deprecated** \| **Index** \| **Help** \| **Weka's home** | |  |
| **PREV CLASS**   **NEXT CLASS** | **FRAMES**    **NO FRAMES**     **All Classes** |  |
| SUMMARY: NESTED \| FIELD \| CONSTR \| METHOD | DETAIL: FIELD \| CONSTR \| METHOD |  |


---


## weka.gui.beans Class TextEvent

```
java.lang.Object
  java.util.EventObject
      weka.gui.beans.TextEvent
```

**All Implemented Interfaces:**: java.io.Serializable

---

``` public class TextEvent extends java.util.EventObject ```

Event that encapsulates some textual information

**Version:**
:   $Revision: 1.4 $

**Author:**
:   Mark Hall

**See Also:**: Serialized Form

---

**Constructor Summary**

`TextEvent(java.lang.Object source,
java.lang.String text,
java.lang.String textTitle)`
  
          Creates a new `TextEvent` instance.


| **Method Summary** | |
| --- | --- |
| `java.lang.String` | `getText()`             Describe `getText` method here. |
| `java.lang.String` | `getTextTitle()`             Describe `getTextTitle` method here. |

**Methods inherited from class java.util.EventObject**

`getSource, toString`

**Methods inherited from class java.lang.Object**

`equals, getClass, hashCode, notify, notifyAll, wait, wait, wait`

**Constructor Detail**

### TextEvent

```
public TextEvent(java.lang.Object source,
                 java.lang.String text,
                 java.lang.String textTitle)
```

:   Creates a new `TextEvent` instance.

    **Parameters:**: `source` - an `Object` value: `text` - a `String` value


**Method Detail**

### getText

```
public java.lang.String getText()
```

:   Describe `getText` method here.

    :   **Returns:**: a `String` value

---


### getTextTitle

```
public java.lang.String getTextTitle()
```

:   Describe `getTextTitle` method here.

    :   **Returns:**: a `String` value


---


|  |  |  |
| --- | --- | --- |
| **Overview** \| **Package** \| **Class** \| **Tree** \| **Deprecated** \| **Index** \| **Help** \| **Weka's home** | |  |
| **PREV CLASS**   **NEXT CLASS** | **FRAMES**    **NO FRAMES**     **All Classes** |  |
| SUMMARY: NESTED \| FIELD \| CONSTR \| METHOD | DETAIL: FIELD \| CONSTR \| METHOD |  |


---
